# Magnitude and associated factors of needle stick and sharps injuries among health care workers in Dessie City Hospitals, north east Ethiopia

**DOI:** 10.1186/s12912-020-00422-0

**Published:** 2020-04-21

**Authors:** Solomon Assen, Mamo Wubshet, Manay Kifle, Tewelde Wubayehu, Berihu Gidey Aregawi

**Affiliations:** 1 Department of Environmental and Occupational Health and Safety, Institute of Public Health, College of Medicine and Health Sciences, University of Gondar, Gondar, Ethiopia; 2 Departments of Public Health, College of Health Sciences, Aksum University, P. O. Box: 298, Aksum, Ethiopia; 3 School of Medicine, Department of Pediatrics, Aksum University, Aksum, Ethiopia

**Keywords:** Needle sticks and sharps injuries, Health workers, Dessie City hospitals, Ethiopia

## Abstract

**Background:**

Work-related exposures to needle stick and sharps accidents are essential reason of infections with blood borne pathogens amongst health care employees and can cause extensive fitness consequences and psychological stress. The aim of this study was to determine the magnitude of needle stick and sharps injuries and associated factors among health care workers in Dessie town hospitals.

**Method:**

This institution-based cross-sectional study was conducted from March 21–April 21/2015, amongst health care people in Dessie city hospitals.

Data have been collected by a structured and pre-tested questionnaire. The study included 438 health care employees who had been selected through the use of a simple random sampling technique. The gathered data have been checked, coded and entered to EPI-info version 3.5.1 and exported to SPSS model 20 for analysis. Bivariate and multivariate logistic regression analyses have been executed to identify elements related with the structured variable.

**Results:**

From 457 selected study participants, 438 (95.8%) responded to the questionnaire. The magnitude of needle stick and sharps injuries in the last 12 months was 124(28.3%), of which 92(74.2%) was reported by males and the rest 32(25.8%) by females. Being male [AOR: 4.25, 95%CI:(2.43,7.41)],had no safety instructions in the work area [AOR:2.27,95%CI: (1.29,3.97)],had no training on safety and health [AOR:4.92,95%CI:(2.75,8.79)],had ≤5 years work experience [AOR:9.0,95%CI:(4.88,16.60)],recapping of used needle [AOR: 2.63, 95%CI: (1.39, 4.99)] were the variables that significantly associated with needle stick and sharps injuries.

**Conclusion:**

This study showed still a high magnitude of needle stick or sharps among healthcare workers. Therefore, training on work related safety and wellbeing, making safety instructions accessible and avoiding a recap of the needle after use are important to reduce the chance of such injuries among healthcare workers.

## Background

Needle stick or sharps injuries are caused by different types of needles, lancet, surgical scalpel, trocar puncture needle, vacuum tube blood collection needle, broken vial preparation, razors, scissors, etc. during patient cares [1, 2]. Percutaneous exposures to blood and body fluids all through infected needle stick and sharps accidents are the main occupational hazard for morbidity and mortality from infections with blood borne pathogens among health care workers [1]. Needle sticks and sharps injuries (NSSIs) cause a great awesome to the transmission of blood borne pathogens [3]. According to World Health Organization (WHO) report, presentation to sharps within the working environment contributes to 40% of contaminations with Hepatitis B Infection (HBV) and Hepatitis C Infection (HCV) and 2–3% of HIV diseases among health care workers [4]. In developed countries, contact to HBV in health care workers is common, with an amplified risk of at least 3–6 times that of the common populace [4, 5]. In developing countries, this number is estimated to be 6–18 times more; about 40–60% of HBV infectivity in health care workers were attributed to the professional hazard while in developed countries the attributed portion was less than 10% [4]. Particularly, those in sub-Saharan countries, have the top incidences of occupational exposures [6]. Occupational NSSIs affect the quality of health care services other than the health care providers [1]. Health care workers experience rigorous emotional distress, fear, and anxiety which lead them to occupational and behavioral changes [7, 8]. From thirty five million health care workers, three million experience NSSIs every year worldwide [9], with a top incidence of these injuries being reported from health institutions in several countries [10, 11]. Studies conducted in different nations showed that the level of NSSIs among health workers was exceptionally high [12]. Approximately, 1.2 million healthcare staff are exposed to needle stick injuries every year in Europe [13]. A study in Germany university hospital showed that 31.4% of health care workers sustained at least one needle stick injury in one year [8] while in Greek the overall injury rate of health care was 2.4% per year out of the total full-time employees engaged in patient-related activities [14]. Studies in health care workers in Iran, Malaysia, Saudi Arabia showed a prevalence rate of 24.9, 74, and 39.4% respectively [12, 15, 16]. Similar studies in Nigeria, Uganda, and Kenya showed a one-year prevalence of needle stick/sharps object injuries were 18.5, 32.4 and 20.2%, respectively [17–19].

In Ethiopia, only a few studies have been done on the prevalence of needle stick/sharps injuries [20, 21] and even the findings of these studies did not address the magnitude of NSSIs well among all parts of health care workers (HCWs). So, this study was conducted to assess the magnitude and associated factors of needle stick and sharps injuries, which may have a contribution in the development of prevention strategies.

## Methods

### Study design, period, and study area

Institution-based cross sectional quantitative study was conducted among HCWs in Dessie City Hospitals from March 21 to April 21/2015. Dessie is the capital city of South Wollo Zone and it has 10 sub city wards and 6 rural wards with a total population of 188,519 based on the 2007census. There were a total of 774 health care workers assigned to provide with health services to the community in the two governmental and three private hospitals.

The distribution of study populations in the two governmental Hospitals was 51 physicians, 239 nurses, 55 midwives, 21 health officers, 29 laboratory technologists, 11 anesthetists, and 54 cleaners. Similarly, there were 37 physicians, 102 nurses, 34 midwives, 28 health officers, 22 laboratory technologists, 7 anesthetists, and 54 cleaners in the three private hospitals.

### Study population

All health care workers (physicians, nurses, lab technicians/technologists, cleaners, midwives, health officers, and anesthetists) that worked for 1 year and above in the two governmental and three private hospitals and that participated in screening, diagnosis, treatment, and follow-up of patients with the assistance of needle and sharps medical equipment, and handling of waste disposal were included in the study.

### Exclusion criteria

Resident doctors and students were not participants of the study**.**

### Sample size determination and sampling procedure

Four hundred fifty-seven participants were the sample size of this study which was calculated using a single population proportion formula, 22.2% [22] previous study of NSSIs from East Gojjam, 4% margin of error, 95% confidence interval, and 10% non-response rate.

Based on the assumption that the participants are homogeneous across the hospitals, but heterogeneous within job category with respect to the exposure of needle stick and sharps injuries, the 457 samples were proportionally allocated to each hospital and selected using lottery method from the lists of health care workers. The calculated sample size was proportionally distributed to the five hospitals based on the number of health care works. We used the list of health care workers from the monthly payroll of the five hospitals as a sampling frame. Then, a simple random sampling technique (lottery method) was used to select the study participants.

### Operational definition

Healthcare workers are workers in hospitals (physicians, nurses, lab technicians/technologists, cleaners, midwives, health officers, and anesthetists) who do have contact with syringes, needles and other sharps materials due to their duties.

Safety instructions are written information in the workplace about safe work practices like the prohibition of recapping of needles, disposing of sharps immediately after use in designated sharp containers, engaging the temporary safety locking mechanism on sharps waste disposal box when not in use, and sealing and disposing of sharps containers when full of its three quarter.

OHS training is an awareness program about the different hazards and risks in the working environment, safe working procedure, and infection prevention and safety policy of the organization.

### Data collection procedures

The questionnaire was developed based on related literature (Additional file 1) and had socio-demographic, behavioral, environmental, NSSIs variables. The questionnaire was prepared in English and translated to Amharic. Then, it was translated back to English to keep its consistency.

A pretest was done in Dessie health station on 42 health care workers, and necessary corrections were made based on the result of the pretest. Training was given for 2 days for the 2 supervisors (BSC nurses) and five data collectors (diploma nurses). Data were collected by interviewing health care workers, and the gathered data were checked each day by SA and supervisors.

### Data processing and analysis

Data were checked, coded and entered to the EPI-info model 3.5.1 and exported to SPSS (Statistical Package for Social Science) model 20 for analysis. Bivariate logistic regressions were fitted to identify factors associated with needle stick and sharps injuries. Only variables reached a *p*-value≤0.2 in the bivariate logistic regression analysis had been included in the multivariate logistic regression model to check the confounding effects. Finally, variables that had p-value< 0.05 were considered as having significant association with needle stick and sharps injuries, and results were presented with an odds ratio (OR) and 95 % confidence interval (CI).

## Results

### Socio-demographic characteristics

From 457 selected study participants, a total of 438 (95.8%) health care workers, 245 (55.9%) males and 193 (44.1%) females responded. These participants were within age range from 22 to 59 years and mean (±SD) 33.56 ± 6.41 years. The majority 214 (48.9%) were Orthodox followed by Muslim 160 (36.5%), Protestant 58 (13.2%), and Catholic 6 (1.4%). The socio-demographic characteristics of the respondents were summarized in Table 1.

**Table 1:** Socio demographic characteristics of health care workers in Dessie hospitals (*n* = 438)

Variables	Number	Percent
Sex		
Male	245	55.9
Female	193	44.1
Age (in years)		
18–29	142	32.4
30–44	260	59.4
≥45	36	8.2
Educational level		
≤8grade	38	8.7
9-12grade	28	6.4
Diploma	188	42.9
Degree and above	184	42.0
Marital status		
Single	117	26.7
Married	270	61.6
Divorced and widow	51	11.6
Job category		
Laboratory technologist	29	6.6
Nurse	198	45.2
Physician	64	14.6
Cleaner	65	14.8
Others**	82	18.7
Work experience (in years)		
<5	259	59.1
≥5	179	40.9
Monthly salary (ETB)		
≤1400	66	15.1
1401–3550	209	47.7
3551–5000	99	22.6
>5000	64	14.6

Note: ETB = Ethiopian Birr

Others** = midwives, health officers, anesthetists

### Work environment characteristics

Two hundred sixty-eight (61.2%) and 143 (32.6%) health care workers reported that their workplace had no safety instruction and work guidelines respectively. Similarly, 263 (60%) did not take occupational health and safety training (Table 2).

**Table 2:** Characteristics of the working environment of health care workers in Dessie hospitals (*n* = 438)

Variables	Number	Percent
Presence of safety instructions at workplace		
Yes	182	41.6
No	256	58.4
Presence of work guidelines at workplace		
Yes	295	67.4
No	143	32.6
Getting training on occupational health and safety		
Yes	202	46.1
No	236	53.9
Working hours per week		
<48	173	39.5
>48	265	60.5
Presence of safety boxes at the workplace		
Yes	267	61
No	171	39
Have written protocol for reporting injury		
Yes	54	12.3
No	384	87.7

### Magnitude of needle stick and sharps injuries

From a total of 438 health care workers, 124 (28.3%) faced needle stick and sharps injuries in the last 12 months. Around 117 (94.4%) of the injured health care workers have got injured once and 7 (5.6%) twice and more since the last 12 months. Similarly, 42 (9.6%) health care workers faced needle stick and sharps injure in the last 3 months; from these, 39 (92.9%) got injured once and 3 (7.1%) twice and above. Parts of the body with the highest frequency of NSSIs were fingers 56 (45.2%), while the most frequent 41 (33.1%) cause of NSSIs was syringe needles (Table 3).

**Table 3:** Distribution of NSSIs by parts of the body, needles/sharps that caused the injury and types of injury sustained among injured health care workers in Dessie hospitals (*n* = 124)

Variable	Number	Percent
Parts of the body injured		
Finger	56	45.2
Palm	36	29
Hand	32	25.8
Type of sharps that caused injury		
Syringe needle	41	33.1
Suture needle	22	17.7
An intravenous cannula (catheter)	22	17.7
Lancet insulin needle	13	10.5
Scalpel blade	10	8.1
Butterfly needle	8	6.5
Glass item	6	4.8
Others*	2	1.6
Types of injury sustained		
Superficial injury (skin without bleeding)	50	40.3
Slight skin penetration (with bleeding)	64	51.6
Deep injury	10	8.1

Note: Others* = scissor, blade

In this study, 80(40.4%) nurses, 17 (26.2%) cleaners, and 7 (24.1) laboratory technologists reported sustained needle stick and sharps injury in the last 12 months. Concerning working department, 15 (53.6%) of health care workers in surgical wards, 14 (53.8%) in the operation room, 3 (50.0%) in injection and dressing room and 18 (34.6%) in the delivery room reported that they had faced NSSIs in the last 12 months (Table 4).

**Table 4:** Prevalence of NSSIs by job category and working departments among health care workers in Dessie hospitals in the last 12 months (*n* = 438)

Variables	Have in the NSSIs	
	Yes	No
Job category		
Laboratory technologist	7(24.1)	22(75.9)
Nurse	80(40.4)	118(59.6)
Physician	7(10.9)	57(89.1)
Cleaner	17(26.2)	48(73.8)
Others**	13(15.9)	69(84.1)
Working department		
Emergency/OPD	6(15.4)	33(84.6)
Pediatric ward	14(28)	36(72)
Delivery room	18(34.6)	34(65.4)
Laboratory room	6(18.8)	26(81.2)
Operation room	14(53.8)	12(46.2)
Gynecology ward	10(29.4)	24(70.6)
Medical ward	10(18.2)	45(81.8)
Injection and dressing room	3(50.0)	3(50)
Surgical ward	15(53.6)	13(46.4)
Orthopedic ward	3(23.1)	10(76.9)
Ophthalmology room	1(4.5)	21(95.5)
Dental room	2(15.4)	11(84.6)
Cleaner	22(32.4)	46(67.6)

Others* = Health Officers, Midwives, Anesthetist

**Fig. 1:**
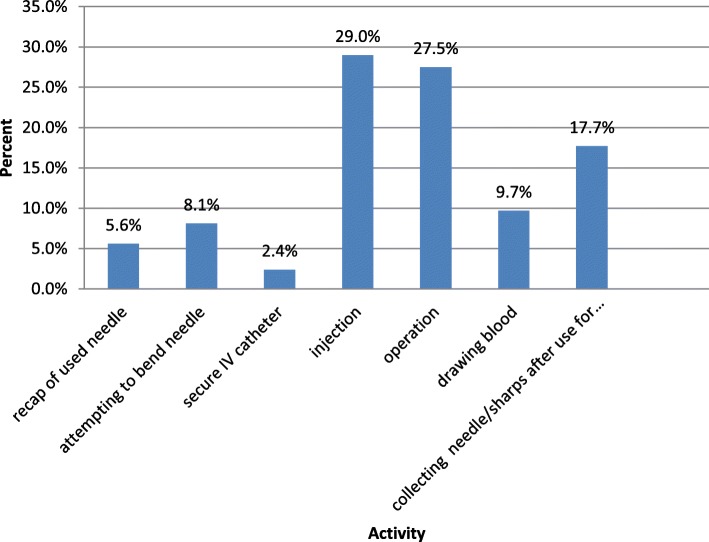
Activities Performed by Health Care Workers at the Time of Injury in Dessie Hospitals, North East Ethiopia

The majority of the injuries 36(29%) occurred during injection, followed by operation 34 (27.5%) and collecting needle and sharps after use 22(17.7%) (Fig. 1). The highest number 61(49.2%) of the NSSIs occurred on Monday (Table 5).

**Table 5:** Days and time of injury of health care workers in Dessie hospitals (*n* = 124)

Variables	Injured study participants (*n* = 124)	
	Frequency	%
Days of injury		
Monday	61	49.2
Tuesday	7	5.6
Wednesday	13	10.5
Thursday	9	7.3
Friday	5	4
Saturday	1 2	9.7
Sunday	5	4
I do not remember	12	9.7
Time of injury		
Morning	55	44.4
Afternoon	33	26.6
Evening	13	10.5
Night	18	14.5
I do not remember	5	4

### Behavioral and practical characteristics

Out of the total (438) HCWs, 254(58%) were concerned about needle stick/sharps injuries in their work environment and 276(63%) considered needle stick/sharps injuries were unavoidable. From 124 injured HCWs, 68(54.8%) did not report their injuries and the reasons for not reporting were lack of support from management 27 (39.7%), and not considering reporting as important 19 (27.9%). About 313 (71.5%) participants recap the needle after use and only 51 (11.6%) of the respondents did not use personal protective equipment (PPE) during their working procedure. The most commonly mentioned reason for not using PPE was discomfort when used 24 (47.1%) followed by a lack of PPE 14 (27.5%).

### Factors associated with needle stick and sharps injuries

To assess the relative effect of socio-demographic, environmental and behavioral factors on needle stick and sharps injury logistic regression was done.

In the bivariate logistic regression analysis, NSSI was associated significantly with sex, having safety instruction, OSH training, work experience, working hours/week, recapping of the used needle, drinking of alcohol and chewing khat. However, finally, sex, safety instruction in their work area, OSH training, work experience and the recap of used needles remain significantly associated in the multivariate logistic regression analysis.

Being males [AOR: 4.25, 95%CI: (2.43, 7.41)], not having safety instruction in their work area [AOR: 2.27, 95%CI: (1.29–3.97), not having training on OSH [AOR: 4.92, 95% CI: (2.75, 8.79)], having work experience < 5 years [AOR: 9.0, 95%CI: (4.88, 16.60)] and recapping of used needles [AOR: 2.63, 95%CI: (1.39, 4.99)] were higher risk of getting NSSIs compared to their counterparts (Table 6).

**Table 6:** Bivariate and multivariate logistic regression analysis of potential factors associated with NSSIs among healthcare workers in Dessie hospitals (*n* = 438)

Variables	NSSIs		COR (95% CI)	AOR (95% CI)
	Yes	No		
Sex				
Male	92	153	3.03(1.91,4.79)***	4.25(2.43,7.41)***
Female	32	161	1	
Safety instruction				
No	85	171	1.82(1.18,2.83)**	2.27(1.29,3.97)**
Yes	39	143	1	
OSH training				
No	86	150	2.47(1.59,3.85)***	4.92(2.75,8.79)***
Yes	38	164	1	
Work hours/week				
>48	87	178	1.80(1.15,2.81)**	1.57(0.92,2.68)
≤48	37	136	1	
Work experience				
<5 year	100	159	4.06(2.47,6.68)***	9.0(4.88,16.60)***
≥5 year	24	155	1	
Recap of used needle				
Yes	99	214	1.85(1.12,3.05)*	2.63(1.39,4.99)**
No	25	100	1	
Drink alcohol				
Yes	28	37	2.18(1.27,3.76)**	1.30(0.64,2.65)
No	96	277	1	
Chew khat				
Yes	40	62	1.94(1.21,3.09)**	1.68(0.92,3.06)
No	84	252	1	

Note: * Significant at *p* < 0.05, ** Significant at *p* < 0.01, *** Significant at *p* < 0.001

## Discussions

### Magnitude of needle sticks and sharps injuries

The prevalence of NSSIs among healthcare workers in the last 12 months was 124(28.3%), which is relatively similar to studies conducted in Awassa and Bahir Dar [20, 23]. But it is high compared to studies done in East Gojjam, Ethiopia [22], Kenya [19] & Nigeria [17]. The possible difference in the proportion of injury could be the socio-demographic/economic status, and cultural characteristics of study participants.

Parts of the body with the highest frequency of NSSIs were fingers 56(45.2%). This result was similar to a study conducted in Saudi Arabia [24]. This could be linked to the fact that the fingers are involved in the handling of needles and other sharps, in recapping of the needles, suturing and setting intravenous lines.

In this study, the majority of HCWs who reported to have sustained needle stick and sharps injury were nurses 80(40.4%), and this is in agreement with a study done in Pretoria [25], and much higher than studies done in Kenya [19] and Nigeria [17]. The possible difference may be due to the ratio of nurse to people is not proportional as set by the WHO. Another explanation that may account for the high percentage of the injuries reported by nurses was the fact that most 198(45.2%) of study participants were nurses.

There are also a greater proportion of 17 (26.2%) injuries reported by cleaners compared to other health care workers. This could be due to the reason that they are usually people from the lower socioeconomic status and lower educational background; no visible programs are available to teach them about the risks of occupational exposure to NSSIs.

The most frequent 41 (33.1%) cause of NSSIs was syringe needles, which was in line with the findings of other studies [15, 24, 26]. This might be due to the fact that syringe needles have been used in every department of the health care facilities unlike that of other sharps which have been used only in a few departments.

The majority of the injuries, 36(29%) occurred during injection, followed by operation 34 (27.5%), and collecting needles and sharps after use 22(17.7%).

The highest number 61(49.2%) of the NSSIs occurred on Monday. The possible explanation for this might be linked to high patient flow and a heavier workload on Monday. However, in Pakistan Public Sector Tertiary Care Hospitals, most injuries among healthcare workers occurred on Saturday [27]. This difference could be explained by the setup and culture difference between the two countries.

As the multivariate logistic regression analysis indicated, male are 4.25 times more likely to get exposed to NSSIs than females. Similar finding in a study undertaken in Malaysia indicated that there was a significant association between sex of the worker and the occurrence of sharps and needle stick injury among HCWs [15]. This may be since females are better in safety precautions compared to males.

Those respondents had no safety instruction in their work area were significantly at higher risk of getting NSSIs compared to those who had safety instruction. This finding was supported by the study conducted on needle stick injuries among doctors working in a tertiary care hospital of Karachi [10].

Respondents whoever had no training on OSH were significantly at higher risk of getting NSSIs compared to those whoever had training on OSH. This finding was supported by a study done on the prevalence of needle stick injuries among HCWs in a tertiary care hospital in Delhi India [5].

In this study, the HCWs who had work experience < 5 years were significantly at higher risk of getting NSSIs compared to≥5 years. The finding was supported by a study taken on in turkey; it confirmed that the risk of needle stick and sharps injuries were higher among health care workers of experience < 5 years [3]. This might be due to the fact that less attention has been given to the exposure of NSSIs in less experience HCWs.

In the study area, HCWs who recapped used needles indicated that they were 2.63 times more likely to get injured compared to their counterparts. This finding was supported by the study conducted in the southern part of Ethiopia, where half of the HCWs recapped needle after used [28]. This may be due to inappropriate needle handling practices like recapping, disposing of needles, and because of insufficient training of HCWs or their refusal to follow the correct procedures.

The study could have limitations. Since participants have been asked a 1 year exposure experience, there might be recall bias and limitations of data collection due to potential participants’ fear.

## Conclusions

The magnitude of needle stick or sharps accidents among HCWs in the hospitals in which the study took place was 28.3%. This showed still a high magnitude of NSSIs among healthcare workers, which might be an indication that the risk of health care workers toward blood borne infections, including HIV/AIDS and different hepatitis is getting increased. Lack of safety instructions, insufficient OSH services, like training on occupational health hazards and safety issues, an absence of a written protocol for reporting needle stick and sharps injuries may have contributed to expose health care workers to the risk of NSSIs. Therefore, Putting in a framework that encourages health care workers to wear PPE through accessing and supplying standard materials to the services delivered in the health care facility, training on OSH, making safety instruction available, and avoiding a recap of the needle after use are important to reduce the risk of such injuries among HCWs. Further investigation is required to determine the actual occurrence of needle stick and sharp injury exposure, and the type of disease they would acquire.

## Supplementary information

**Additional file 1.** English Version Questionnaire to Collect Data on the Magnitude and Associated Factors of Needle Stick and Sharps Injuries among Health Care Workers in Dessie City Hospitals, North East Ethiopia

## Data Availability

The datasets used and/or analyzed during the current study available from the corresponding author on reasonable request.

## Abbreviations

AOR: Adjusted Odds Ratio
CDC: Center for Diseases Prevention and Control
CI: Confidence Interval
COR: Crude odds ratio
HBV: Hepatitis B virus
HCB: Hepatitis C virus
HCWs: Health care workers
HIV: Human Immunodeficiency Virus
IV: Intra-venous
NSI: Needle stick injury
NSSIs: Needle sticks and Sharps injuries
PPE: Personal Protective Equipment
SPSS: Statistical Package for Social Science
SPs: Standard Precautions
WHO: World Health Organization
