# Supplementary material for: Magnitude and associated factors of needle stick and sharps injuries among health care workers in Dessie City Hospitals, north east Ethiopia
Source: BMC Nurs. 2020 Apr 21;19:31. doi: 10.1186/s12912-020-00422-0 (PMC7171769; doi:10.1186/s12912-020-00422-0)
Supplement: Supplementary file 1 — Additional file 1. English Version Questionnaire to Collect Data on the Magnitude and Associated Factors of Needle Stick and Sharps Injuries among Health Care Workers in Dessie City Hospitals, North East Ethiopia [file 12912_2020_422_MOESM1_ESM.docx]

**Additional file 1: English version Questionnaire**

Magnitude and Associated Factors of Needle Stick and Sharps Injuries among Health Care Workers in Dessie City Hospitals, North East Ethiopia

**Questionnaire identification number_________________**

Name of the hospital ____________________

Name and Signature of the data collector who sought the consent______________

Date of interview_________ time started __________ time completed___________

Checked by supervisor: Name and signature_______________________________

date__________________, Identification No

**Section one: Socio demographic information**

| **No** | **Question** | **Possible Responses** | code |
| --- | --- | --- | --- |
| 101 | Name of hospital | 1.Dessie referral hospital 2.Selam hospital  3.Boru hospital 4.Bati hospital 5.Ethio hospital |  |
| 102 | Sex | 1. Male 2. Female |  |
| 103 | Age | Years |  |
| 104 | Religion | 1. Orthodox 2.Catholic 3.Protestant 4. Muslim  5. Others Specify___________________ |  |
| 105 | Marital status | 1.Single 2.Married 3.Divorced 4.Widowed 5.Cohabited |  |
| 106 | Educational level | 1. Illiterate 2. Read and Write 3. 1-4 Grade  4. 5-8Grade 5. 9-10 Grade 6.11-12 Grade 7.technical and vocational 8. Diploma 9.degree and above |  |
| 107 | Your job category | 1.laboratory technician 2.anesthetist 3.gynecologist 4.clinical nurse 5.midwife nurse 6.general practitioner 7.internist 8.health officer 9.pediatrician  10. Surgeon 11.cleaners  12. other please specify |  |
| 108 | Work experience | in month/years |  |
| 109 | monthly salary | ETH BIRR/month |  |

**Section two: Information Related to Work Environment**

| **No** | **Question** | | **Possible Responses** | **skip** |
| --- | --- | --- | --- | --- |
| 201 | Are there safety instructions at your work environment? | | 1. Yes 2. No |  |
| 202 | Are there work procedures /guidelines at your workplace? | | 1. Yes 2. No |  |
| 203 | Have you ever had training on occupational health and safety? | | 1. Yes 2. No |  |
| 204 | Have you ever participated training program about infection prevention caused by needle stick/sharp injury? | | 1. Yes 2. No |  |
| 205 | Is there a written protocol for reporting needle stick and sharp injuries in your organization? | | 1. Yes 2. No |  |
| 206 | What shift had you most often been assigned to do your practice in the last 12 months?   1. Day shift 2. Evening shift 3. Night shift | | |  |
| 207 | The average number of hours worked per week? | |  |  |
| 208 | In which department do you work? 1. Emergency/OPD 2.Pediatric Ward 3.delivery room 4.Laboratory Room 5.Operation Room 6.Gynecology Ward  7. Medical Ward 8. Injection and dressing room 9. Surgical Ward 10. Orthopedic Ward  11. Ophthalmology Room 12. Dental Room 13.Other(specify) | | |  |
| 209 | Are there sharp containers (safety box) available at your workplace? | 1. Yes 2. No | | If  No, skip  toQ211 |
| 210 | If yes to Q 209, are they accessible and in use? | 1. Yes 2. No | |  |
| 211 | Were you supervised by safety officer or concerned bodies? | 1. Yes 2. No | |  |
| 212 | If yes Q. number 211, how much is the frequency? | /week/month/year | |  |

**Section Three: needle stick and Sharp injuries characteristics**

| No | Question | Possible Responses | Escap |
| --- | --- | --- | --- |
| 301 | Had you got needle stick /Sharp injury in the last 3 months? | 1. Yes 2. No | If No, skip to Q303 |
| 302 | If yes to Q301, how many times? | 1. Once 2. 2-3 times 3. 4 & more |  |
| 303 | Had you got needle stick / sharp injury in the last 12 months? | 1. Yes 2. No | If No, skip to Q305 |
| 304 | If yes to Q303, how many times? | 1. Once 2. 2-3 times 3. 4 & more |  |
| 305 | What Part of the body injured? | 1. Palm 2.fingers 3. Hand  4. Leg 5. others please specify |  |
| 306 | What were you doing at the time of injury? 1.Recapping of used needle 2.Attempting to bend the needle  3. Opening the needle cap 4. Dressing and injection 5.Operation 6.Drawing blood  7.collecting needle and syringe after use for disposal 8.other(specify) | |  |
| 307 | Type of items caused the injury? 1. Syringe needle 2.Lancet insulin needle 3. Scalpel blade  4. Intravenous cannula (catheter) 5.suture needle 6.butterfly needle 7.Glass item 8.Scissor 9.Blade 10.Other Sharp(Specify) | |  |
| 308 | Day of injury occurred? 1. Monday 2.Tuesday 3. Wednesday 4. Thursday  5. Friday 6.Saturday 7. Sunday 8. I do not remember | |  |
| 309 | What time of injury occurred? 1. In the morning 2. In the afternoon 3. In the evening  4. In the night 5. I do not remember | |  |
| 310 | Did you receive medical care for your injury? | 1. Yes 2. No |  |

**Section four: Information on workers’ behavior,**

| No | Question | | Possible Responses | | Code |
| --- | --- | --- | --- | --- | --- |
| 401 | Are you concerned about needle stick/sharps injury in your workplace? | | 1 .yes 2. No | |  |
| 402 | Do you consider needle/sharp injuries are avoidable? | | 1. Yes 2.No | |  |
| 403 | Did you report your injury? | | 1.yes 2.No | | If No skip to Q405 |
| 404 | If yes toQ403, to whom you report it? | | 1.To immediate supervisor  2.To my colleague  3.Others, (specify) | |  |
| 405 | If No to Q406, what is the reason for not reporting 1.Lack of support by management 2.Fear of stigma/discrimination 3.Unaware of reporting procedure  4. Reporting is too time consuming 5. I emphasized to patient care  6. I thought I might be blamed or in trouble 7. I did not think it was important  8. I thought the source patient was low risk for HIV 9.Others (specify) | | | |  |
| 406 | Do you recap needles after use? 1. yes 2.NO | | | |  |
| 407 | How do recap needles after use? | | | 1.Using single hand  2.Using two hands |  |
| 408 | Do you use any personal protective equipment? | | | 1. Yes 2. No | If No, skip to Q410 |
| 409 | If yes to Q408, what type of PPE? (you can choose more than one) 1. Apron 2. Utility (double glove) 3. Head cover 4. Boots/shoe 5. Eye protectors/Goggle 6. mask 7. examination glove 8.gown 9.Others(specify) | | | |  |
| 410 | If No Q 408, what are your reasons for not using personal protective equipment?  1. Lack of protective equipment. 2. Lack of safety and health education.  3.Discomfort during use 4.Decrease work performance  5.Create safety and health hazards 6.Other (specify) | | | |  |
| 411 | Do you use /drink alcohol? | 1. Yes 2. No | | | If No, skip to Q413 |
| 412 | If yes to Q No.411 often? | 1. Everyday 2. 1-3 days/week  3. Occasionally | | |  |
| 413 | Do you chew khat? | 1.Yes 2. No | | | If No,skip to Q415 |
| 414 | If yes to Q No.413 how often? | 1.Every day 2. 1-3 days/week  3. Occasionally | | |  |
| 415 | Do you smoke Cigarette | 1.Yes 2. No | | |  |
| 416 | If yes to Q No.415 how often? | 1. Every day 2. 1-3 days/week  3. Occasionally | | |  |

I have completed my questions, thank you for your time and cooperation.
